# The *MUC5B* promoter variant results in proteomic changes in the nonfibrotic lung

**DOI:** 10.1172/jci.insight.189636

**Published:** 2025-06-17

**Authors:** Jeremy A. Herrera, Mark Maslanka, Rachel Z. Blumhagen, Rachel Blomberg, Nyan Ye Lwin, Janna Brancato, Carlyne D. Cool, Jonathan P. Huber, Jonathan S. Kurche, Chelsea M. Magin, Kirk C. Hansen, Ivana V. Yang, David A. Schwartz

**Affiliations:** 1 Division of Pulmonary Sciences and Critical Care Medicine, and; 2 Department of Biochemistry and Molecular Genetics, University of Colorado Anschutz Medical Campus, Aurora, Colorado, USA.; 3 Center for Genes, Environment and Health, National Jewish Health, Denver, Colorado, USA.; 4 Department of Bioengineering, and; 5 Department of Biomedical Informatics, University of Colorado Anschutz Medical Campus, Aurora, Colorado, USA.; 6 Rocky Mountain Regional Veterans Administration Medical Center, Aurora, Colorado, USA.; 7 Department of Pediatrics,; 8 Department of Pathology, and; 9 Department of Microbiology and Immunology, University of Colorado Anschutz Medical Campus, Aurora, Colorado, USA.

**Keywords:** Genetics, Pulmonology, Fibrosis, Proteomics

## Abstract

The gain-of-function *MUC5B* promoter variant is the dominant risk factor for the development of idiopathic pulmonary fibrosis (IPF). However, its impact on protein expression in both nonfibrotic control and IPF lung specimens has not been well characterized. Utilizing laser capture microdissection coupled to mass spectrometry, we investigated the proteomic profiles of airway and alveolar epithelium in nonfibrotic controls (*n* = 12) and IPF specimens (*n* = 12), stratified by the *MUC5B* promoter variant. Through qualitative and quantitative analyses, as well as pathway analysis and immunohistological validation, we have identified a distinct MUC5B-associated protein profile. Notably, the nonfibrotic control alveoli exhibited substantial MUC5B-associated protein changes, with an increase in IL-3 signaling. Additionally, we found that epithelial cells overlying IPF fibroblastic foci clustered closely to alveolar epithelia and expressed proteins associated with cellular stress pathways. In conclusion, our findings suggest that the *MUC5B* promoter variant leads to protein changes in alveolar and airway epithelium that appear to be associated with initiation and progression of lung fibrosis.

## Abstract 2

The MUC5B promoter variant results in spatial proteomic changes that may be associated with initiaiton and progression of lung fibrosis.

## Introduction

Usual interstitial pneumonia (UIP) is a broad category of fibrotic lung diseases that have a poor clinical outcome. Idiopathic pulmonary fibrosis (IPF), the most severe form of lung fibrosis, is defined radiographically and pathologically as UIP (1). Although IPF is traditionally considered idiopathic, emerging literature indicates that genetic factors account for at least 30% of the risk of developing IPF (2–5), and the *MUC5B* promoter variant is responsible for approximately 50% of the genetic risk of IPF (3). Thus, understanding the spatial lung proteome in the context of the *MUC5B* promoter variant should help us decipher critical elements of protein biology in IPF.

The mechanism by which the *MUC5B* promoter variant drives lung fibrosis is an active area of research. It is proposed that ectopic expression of *MUC5B* in epithelial cells (alveolar type II and mucin-producing club cells) causes homeostatic endoplasmic reticulum (ER) stress, yielding a vulnerable lung epithelium requiring a secondary injury to initiate a fibroproliferative response (6). Consequently, we predict that the *MUC5B* promoter variant will have the largest proteomic impact on unaffected lung epithelium.

We previously developed laser capture microdissection coupled to mass spectrometry (LCM-MS) for formalin-fixed and stained lung tissue (7). This method allowed us to determine the proteomic profiles of the 2 lesions of UIP/IPF, the honeycomb cyst (8) and fibroblastic foci (FF) (9). Considering the potential role of the *MUC5B* promoter variant in IPF pathogenesis, we investigated whether characteristic IPF lesions exhibit proteomic changes that are associated with the presence of *MUC5B*. We refer to proteins showing such changes as MUC5B-associated proteins. In addition, we performed LCM-MS to analyze the aberrant basaloid epithelial cells/transitional cells that overlie FF (10–13); herein, we refer to these as epithelia overlying FF. Our specimens were balanced for the *MUC5B* promoter variant in nonfibrotic control and IPF samples.

## Results

### The MUC5B promoter variant is associated with protein changes in nonfibrotic control lungs.

**Figure 1:**
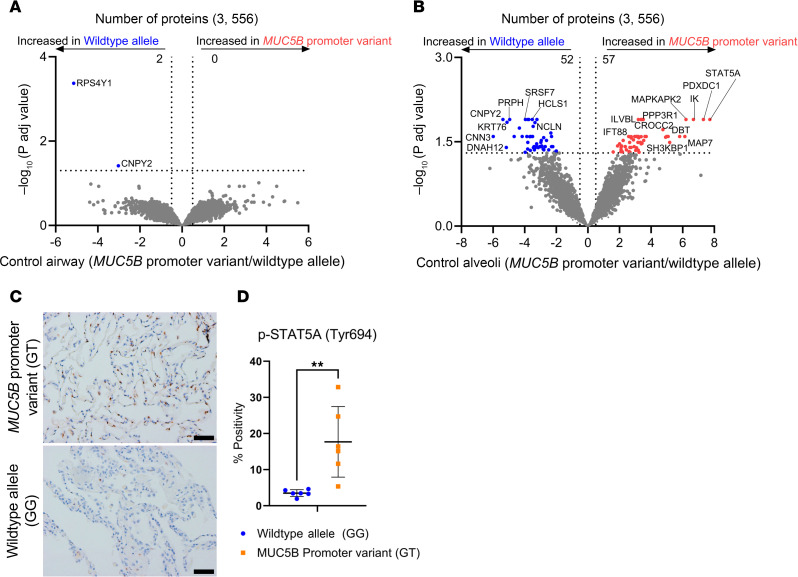
Impact of the *MUC5B* promoter variant on nonfibrotic control lung. (**A** and **B**) Volcano plots comparing the *MUC5B* promoter variant and WT allele in nonfibrotic control (**A**) small airways and (**B**) alveoli, showing the negative natural log of adjusted *P* values (0.05) for each protein. (**C**) Representative images for the IHC against phosphorylated STAT5A at Tyr694 on nonfibrotic control lungs. Scale bars: 50 μm. (**D**) Quantification of **C** showing the percentage of positive cells (*n* = 12, balanced for MUC5B; 5 fields of view per patient with a total of 22,999 counted nuclei). **P < 0.005 by unpaired, 2-tailed *t* test.

We performed LCM-MS analysis on small airways and alveoli from nonfibrotic control specimens (*n* = 12 balanced for the *MUC5B* promoter variant) to determine which proteins are associated with the *MUC5B* promoter variant. Of these specimens, 6 were homozygous for the WT allele (GG) and 6 heterozygous for the *MUC5B* promoter variant (GT). We found that 2 proteins were decreased in nonfibrotic control small airways with the *MUC5B* promoter variant relative to those without: small ribosomal subunit protein eS4, Y isoform 1 (RPS4Y1), and protein canopy homolog 2 (CNPY2) (Figure 1A; a full list of differentially expressed proteins for all *MUC5B* promoter variant comparisons are found in Supplemental Table 1; supplemental material available online with this article; https://doi.org/10.1172/jci.insight.189636DS1).

In addition, we report that the *MUC5B* promoter variant was associated with 109 protein changes in nonfibrotic control alveoli (Figure 1B). The most significantly increased protein was signal transducer and activator of transcription 5A (STAT5A), which functions as a transcription factor. Also increased by the *MUC5B* promoter variant was mitogen-activated protein kinase–activated protein kinase 2 (MAPKAPK2). MAPKAPK2 has been shown to be phosphorylated in IPF epithelial cells and its inhibition reduces bleomycin-induced lung injury (14). Ingenuity Pathway Analysis (IPA) demonstrated that “IL-3 signaling” was the most significantly increased pathway, whereas “cell junction organization” was the most decreased pathway in nonfibrotic control alveoli harboring the *MUC5B* promoter variant (Table 1). At the level of proteomics, MUC5B was not significantly changed in nonfibrotic control alveoli in relation to the *MUC5B* promoter variant. Given that phosphorylation at tyrosine 694 (Tyr694) is a critical site of activation for STAT5A (15), we used immunohistochemistry (IHC) and show marked expression of p-STAT5A (Tyr694) in nonfibrotic control lungs harboring the *MUC5B* promoter variant (Figure 1, C and D). These protein changes in nonfibrotic lungs suggest that the *MUC5B* promoter variant may establish a vulnerable distal lung epithelium.

### The histopathological lesions of IPF uniquely cluster.

**Figure 2:**
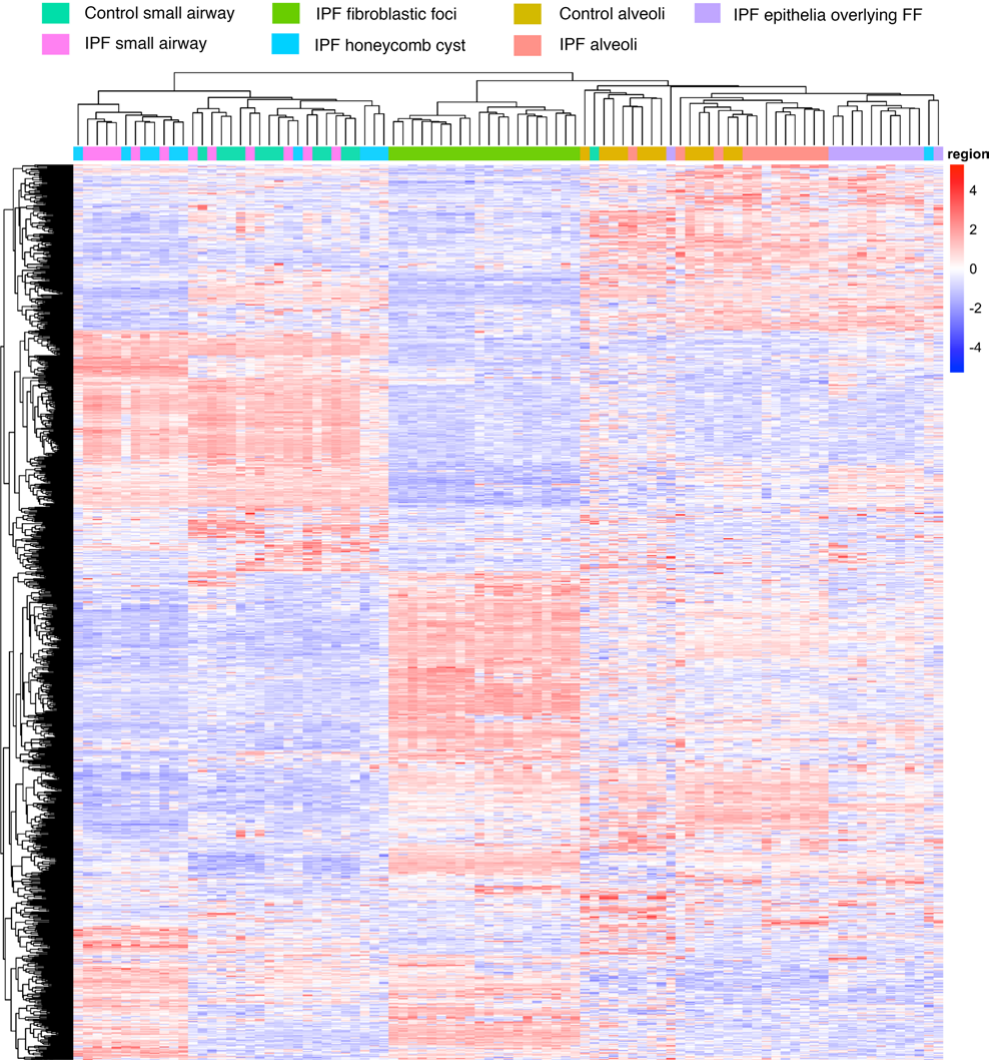
The histopathological lesions of IPF uniquely cluster. A hierarchical cluster analysis based on the top 1,000 variable proteins. For IPF specimens (*n* = 12), we collected 12 small airway, 11 honeycomb cyst, 12 epithelia overlying fibroblastic foci (FF), 12 alveoli, and 12 FF samples from the same 12 IPF specimens. We further collected an additional 8 FF samples (a total of 20 FF samples) from 8 additional IPF specimens. For nonfibrotic control specimens (*n* = 12), we collected 12 small airway and 12 alveoli samples from the same specimen. The samples were balanced for the *MUC5B* promoter variant.

To guide our understanding of IPF at the protein level, we performed hierarchical clustering of our regions of interest (IPF honeycomb cyst, IPF small airways, IPF FF, IPF alveoli, IPF epithelia overlying FF, nonfibrotic control small airway, and nonfibrotic control alveoli) based on the top 1,000 differentially expressed proteins (Figure 2). These regions were histologically confirmed by a pathologist (representative laser-captured images in Supplemental Figure 1). We found that these regions of interest clustered into 3 groups, with some deviations. Firstly, we found that IPF honeycomb cyst clustered with both IPF and nonfibrotic control small airways. We additionally found that the IPF FF samples clustered together. Lastly, we found that the alveolar samples clustered with the IPF epithelia overlying FF.

### Unaffected IPF epithelium differs from nonfibrotic control epithelium, at the protein level.

To understand the protein profiles of IPF airways, we first used a qualitative approach to determine which proteins are present in the airways. We considered a protein present if it was detected in 80% or more of samples within each group. Using this approach, we detected a total of 2,719 airway proteins when grouping IPF honeycomb cyst, IPF small airways, and nonfibrotic control small airways (Supplemental Table 2). Of the 2,719 total airway proteins, 175 were uniquely expressed in the normal-appearing IPF small airways (Supplemental Figure 2A). Gene enrichment analysis of the unique IPF small airway proteins showed that the most significantly upregulated pathways were “anchoring of the basal body to the plasma membrane” and “cilium assembly,” which are pathways involved in ciliogenesis (Supplemental Table 3).

**Figure 3:**
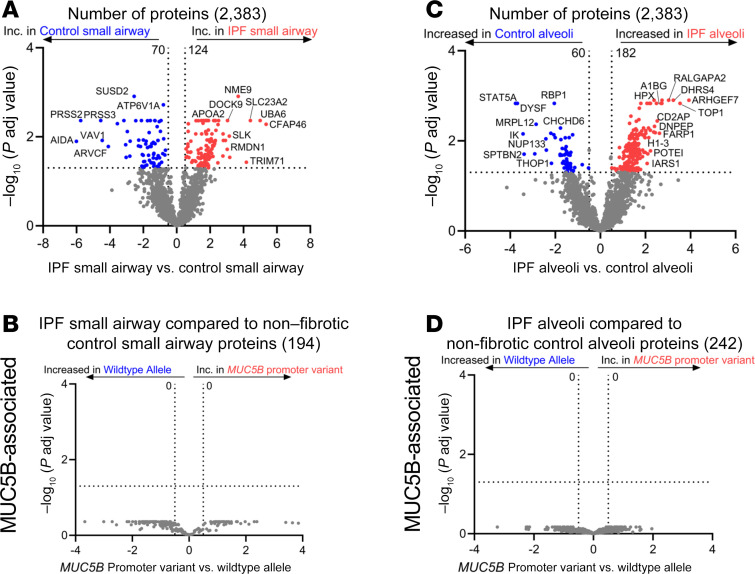
The unaffected epithelia (airway and alveoli) of IPF are abnormal. (**A** and **C**) Volcano plots comparing (**A**) IPF small airway and nonfibrotic control small airways and (**C**) IPF alveoli and nonfibrotic control alveoli, showing the negative natural log of adjusted *P* values plotted against the log_2_(fold change) for each protein. (**B** and **D**) Focusing on the significantly changed proteins from **A** and **C**, we reanalyzed the data for the *MUC5B* promoter variant and show volcano plots for (**B**) IPF small airways and (**D**) IPF alveoli. *n* = 12 per group (6 WT and 6 *MUC5B* promoter variant).

We next performed a quantitative analysis and found 124 significantly increased proteins in IPF small airways and 70 proteins increased in nonfibrotic control small airways (Figure 3A; a full list of differentially expressed proteins for all regions are found in Supplemental Table 4). The most significantly increased protein in IPF small airways was cilia- and flagella-associated protein 46 (CFAP46). Axin interactor, dorsilization-associated protein (AIDA), which has been shown to antagonize the JNK signaling pathway (16), was the most significantly decreased protein. IPF small airway pathway analysis demonstrated an increase in “Rho GTPases activate IQ motif–containing GTPase-activating proteins (IQGAPs)” and “posttranslational protein phosphorylation” (Table 2). IQGAPs regulate many cellular processes, including MAPK signaling pathways (17). These results support the concept that the unaffected small airways in IPF differ from nonfibrotic control small airways, demonstrating elevated levels of components involved in signaling pathways and ciliogenesis. However, none of the 194 significantly changed proteins in IPF small airways were differentially regulated by the *MUC5B* promoter variant (Figure 3B).

To understand the protein profiles of unaffected alveoli in IPF, we performed a qualitative analysis comparing the epithelia overlying FF with IPF and nonfibrotic control alveoli and detected a total of 1,996 proteins (Supplemental Figure 2B). This comparison is based on the observation that the epithelia overlying the FF are positive for alveolar markers (9, 18), and cluster with nonfibrotic control and IPF alveoli based on unsupervised analysis (Figure 2). We then performed a gene enrichment analysis of the 104 unique IPF alveolar proteins and found that “regulation of insulin secretion” and “integration of energy metabolism” were among the mostly robustly enriched pathways.

To understand IPF alveoli in further detail, we quantitatively compared IPF alveoli to nonfibrotic control alveoli and found 242 differentially expressed proteins (Figure 3C). The most significantly increased protein in IPF alveoli was Rho guanine nucleotide exchange factor 7 (ARHGEF7), which is a guanine exchange factor for Rac1 and Cdc42 (19); Cdc42 knockout in alveolar type II cells (AT2) drives periphery-to-center lung fibrosis (20). Pathway analysis of IPF alveoli demonstrated an increase in a variety of pathways involved in translational control (e.g., eukaryotic translation initiation and elongation) (Table 3). Our results confirm that the unaffected IPF alveoli adjacent to FF are biologically abnormal, with increased proteins involved in translational control and metabolism. However, none of the 242 differentially expressed alveolar proteins are regulated by the *MUC5B* promoter variant (Figure 3D).

### Membrane trafficking and remodeling of epithelial adherens junctions define IPF honeycomb cysts.

**Figure 4:**
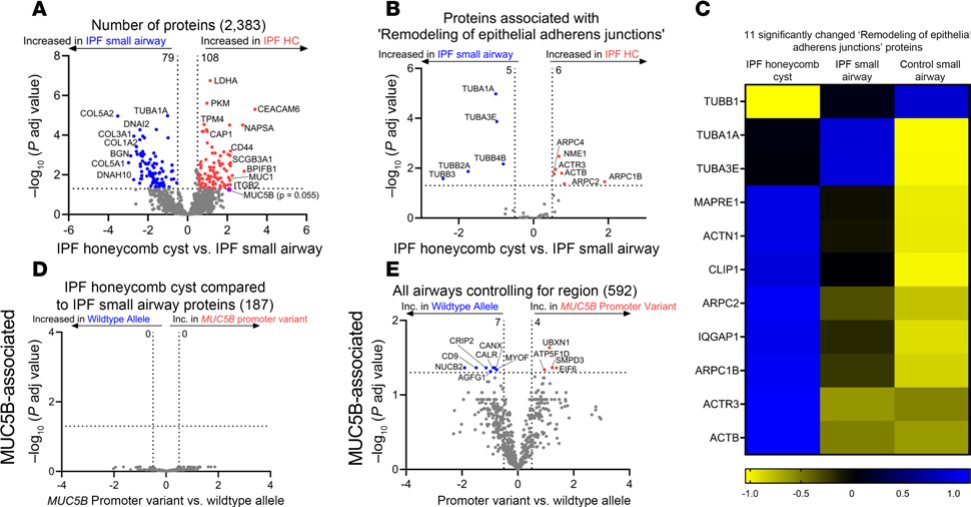
IPF honeycomb cysts are defined by remodeling of epithelial adherens junctions. (**A**) Volcano plot comparing IPF honeycomb cyst (HC) and IPF small airways. (**B**) The subset of proteins associated with “remodeling of epithelial adherens junctions,” showing the negative natural log of adjusted *P* values plotted against the log_2_(fold change) for each protein. (**C**) A heatmap displaying *z* scores for the significantly changed “remodeling of epithelial adherens junctions” proteins, comparing IPF HC, IPF small airways, and nonfibrotic control small airways. (**D** and **E**) Volcano plots comparing *MUC5B* promoter variant and WT allele in (**D**) the subset of significantly changed IPF HC proteins in **A** and (**E**) the subset of all significantly changed airway proteins in IPF and nonfibrotic controls while controlling for region. *n* = 12 per group (6 WT and 6 *MUC5B* promoter variant).

We next focused our analysis on the IPF characteristic lesions: honeycomb cysts and FF. To understand the biology of IPF honeycomb cysts, we first performed a gene enrichment analysis of the 258 uniquely expressed IPF honeycomb cyst proteins (Supplemental Figure 2A). The most significantly enriched pathway was “membrane trafficking,” a secretory membrane system that may reflect increased mucus production in this region. We next performed a quantitative analysis and found 187 significantly regulated proteins in IPF honeycomb cysts when compared with adjacent IPF small airways (Figure 4A). Several mucins or secretory-associated proteins were significantly increased in IPF honeycomb cysts, including MUC5B, MUC1, BPIFB1, SCGB3A1, and NAPSA. Pathway analysis of IPF honeycomb cyst (as compared with IPF small airway) showed enrichment of “remodeling of epithelial adherens junctions” as the most significantly increased pathway, whereas the most decreased pathway was “cilium assembly” (Table 4).

We next focus on the proteins associated with “remodeling of epithelial adherens junctions” when comparing between IPF honeycomb cyst and IPF small airway (Figure 4B) and when comparing nonfibrotic control small airways to IPF airways (Figure 4C). Some notably increased proteins are associated with the actin-related protein 2/3 (ARP2/3) complex (ARPC1B, ARPC2, ARPC4, ACTR3). The ARP2/3 complex is important in generating actin networks to allow for several cellular processes, including motility, membrane trafficking, and endocytosis (21). Actin networks are coupled to the ECM through their adhesive contacts, which have been associated with ECM remodeling. When comparing IPF honeycomb cyst to nonfibrotic control small airway, we found that the most significantly decreased pathways relate to ECM remodeling (collagen degradation, assembly of collagen fibrils and other multimeric structures, and ECM homeostasis) (Supplemental Figure 3). Further research into actin networks, airway cell adhesion, and ECM remodeling are warranted.

**Figure 5:**
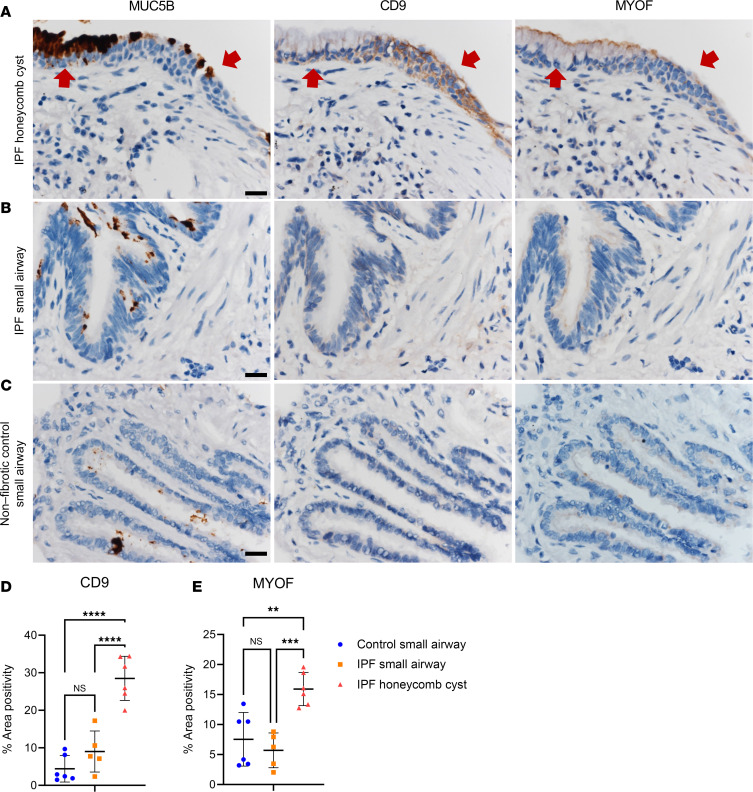
Airway expression of adhesion-associated proteins CD9 and myoferlin. Representative IHC staining for mucin 5B (MUC5B), CD9, and myoferlin (MYOF) in (**A**) IPF honeycomb cyst (red arrows), (**B**) IPF small airway, and (**C**) nonfibrotic control small airway. Scale bars: 20 μm. Quantification of the IHC staining for (**D**) CD9 and (**E**) MYOF positivity per airway (*n* = 6 nonfibrotic control and *n* = 6 IPF specimens). **P < 0.005; ***P < 0.0005; ****P < 0.0001 by 1-way ANOVA.

Interestingly, none of the 187 significantly changed IPF honeycomb cyst proteins were regulated by the *MUC5B* promoter variant (Figure 4D). We next reassessed whether any of the 592 significantly altered airway proteins, from all airway sample types (nonfibrotic control small airway, IPF small airway, and IPF honeycomb cyst), are impacted by the *MUC5B* promoter variant while controlling for region, as previously performed using spatial transcriptomics data (22). We found 11 significantly changed MUC5B-associated proteins (Supplemental Figure 4 and Figure 4E). Consistently with our proteomic results, CD9 and myoferlin (MYOF) were increased in IPF honeycomb cysts (Figure 5, A–E). We next assessed CD9 and MYOF expression in specimens with and without the *MUC5B* promoter variant in IPF specimens (Supplemental Figure 5). We found that the expression in IPF honeycomb cysts was highly variable, which may reflect a limitation of antibody-based approaches as compared with the high sensitivity of MS (23).

### FF are defined by increased translational control.

We next sought to understand the proteomic signature of the IPF FF. A limitation to understanding the biological function of the FF is the lack of a fibroblastic structure in control lungs to compare against. Herein, we compared the FF to adjacent alveolar structures, which had been previously done (9, 24). Proteomic changes may likely be reflective of the varying cell types within the alveoli (alveolar, fibroblast, immune, and vasculature cells) as opposed to FF (predominantly fibroblasts), which may confound downstream interpretations.

In our qualitative analysis, we identified 757 uniquely expressed proteins in IPF FF (Supplemental Figure 2C). Gene enrichment analysis demonstrated pathways involved in translational control (ribosomal scanning and start codon recognition, translation initiation complex, and activation of the mRNA upon binding of the cap-binding complex and eukaryotic initiation factors [eIFs]) and ECM (collagen biosynthesis, collagen formation, and ECM proteoglycans) were enriched in the FF.

**Figure 6:**
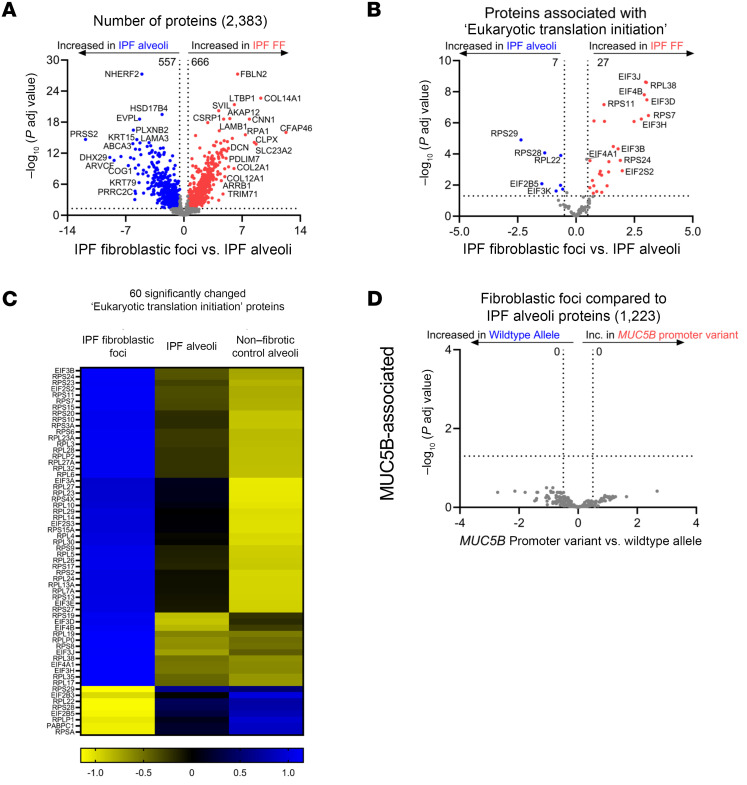
IPF fibroblastic foci (FF) are defined by increased translational control. (**A**, **B**, and **D**) Volcano plots comparing (**A**) IPF FF versus IPF alveoli, (**B**) the subset of proteins associated with “eukaryotic translation initiation,” and (**D**) the subset of significantly changed proteins from **A** reanalyzed for the *MUC5B* promoter variant showing the negative natural log of adjusted *P* values plotted against the log_2_(fold change) for each protein. (**C**) A heatmap displaying *z* scores of the significantly changed “eukaryotic translation initiation” proteins comparing IPF FF, IPF alveoli, and nonfibrotic control alveoli. *n* = 12 per group (*n* = 20 IPF FF, balanced for the *MUC5B* promoter variant).

We next performed a quantitative analysis of the IPF FF as compared to adjacent IPF alveoli (Figure 6A). Increased proteins in the IPF FF are ECM related, such as fibulin-2 (FBLN2), latent TGF-β binding protein 1 (LTBP1), and collagen XIV (COL14A1). The most significantly decreased protein was Na^+^/H^+^ exchange regulatory cofactor NHE-RF2 (NHERF2), a protein primarily expressed in endothelial cells and not fibroblasts (IPF Cell Atlas; https://www.ipfcellatlas.com/). Consistent with our qualitative analysis, pathway analysis of the quantitative data also demonstrated an increase in eukaryotic translation initiation (Table 5). When comparing IPF FF to nonfibrotic control alveoli (Supplemental Figure 6), we additionally found increased translation control, including eukaryotic translation initiation, elongation, and termination. Decreased pathways in IPF FF included negative regulators of translational control, such as PTEN signaling and mTOR regulation.

We next focused on the proteins comprising eukaryotic translation initiation (Figure 6, B and C). The initiation of translation, particularly the binding of the eIF4F complex to the 5′ mRNA cap, is a critical rate-limiting step in the process of translating mRNA into protein (25). The eIF4F complex is composed of eIF4A, -4E, and -4G, with eIF4A showing a significant increase (log_2_ of 0.46) in the IPF FF. Additionally, eIF4B is elevated in the FF and is associated with eIF4A. Prior work has shown that IPF-derived fibroblasts exhibit deranged translational control, and that ECM transcripts are translationally activated by fibroblasts when interacting with pathological ECM (26, 27). In our comparison, we found that the most increased pathway in IPF FF was “collagen biosynthesis and modifying enzymes,” with a *z* score of 4.26. Furthermore, we identified 119 ECM proteins that show significant changes in composition when comparing nonfibrotic control alveoli to IPF FF (Supplemental Figure 6). Thus, the proteomic signature of IPF FF is likely reflective of enhanced translational control that favors fibroblast translation of ECM transcripts. However, none of the 1,223 differentially expressed proteins associated with IPF FF are regulated by the *MUC5B* promoter variant (Figure 6D).

### Cellular stress defines the epithelia overlying the IPF FF.

**Figure 7:**
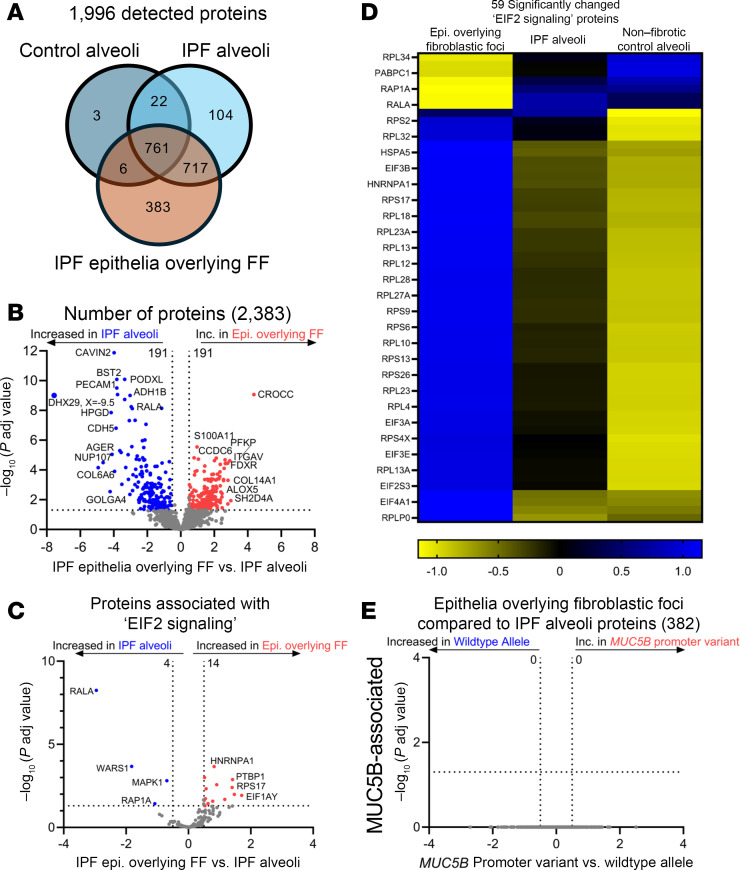
IPF epithelia overlying the fibroblastic foci (FF) are defined by cell stress pathways. (**A**) Venn diagram of detected proteins in the IPF epithelia overlying FF as compared with IPF and nonfibrotic control alveoli. (**B**, **C**, and **E**) Volcano plots comparing (**B**) IPF epithelia overlying the FF and IPF alveoli, (**C**) the subset of proteins associated with “eIF2 signaling,” and (**E**) the subset of significantly changed proteins in **B** reassessed for the *MUC5B* promoter variant, showing the negative natural log of adjusted *P* values plotted against the log_2_(fold change) for each protein. (**D**) A heatmap displaying *z* scores of the significantly changed “eIF2 signaling” proteins comparing IPF epithelia overlying FF, IPF alveoli, and nonfibrotic control alveoli. *n* = 12 per group (6 WT and 6 *MUC5B* promoter variant).

We sought to define the IPF epithelia overlying FF and performed a gene enrichment analysis on the 383 uniquely expressed proteins (Figure 7A and Table 6). We found that the most significantly increased pathway was “cellular response to stress.” This is consistent with a prior report showing that the epithelium lining the FF positively expresses ER stress mediator activation transcription factor 4 and 6 (ATF4 and ATF6, respectively) (28).

To further determine the functionality of the epithelia overlying the FF, we first compared these cells to IPF alveoli (Figure 7B and Table 7). Pathway analysis demonstrated that “processing of capped intron-containing pre-mRNA” and “eIF2 signaling” were the most overrepresented pathways in the epithelia overlying FF. Signaling through eIF2 has been shown to selectively translate mRNAs related to ER stress, such as ATF4 (29). Our proteomic results support recent spatial transcriptomic data showing that the transitional regions of IPF lungs, characterized by enrichment of FF, exhibit elevated “eIF2 signaling” (30). We next focused on the proteins associated with eIF2 signaling (Figure 7, C and D). The most significantly increased protein in the epithelia overlying FF was heterogeneous nuclear ribonucleoprotein A1 (HNRNPA1), a protein that is sequestered to stress granules upon cellular stress (31). Other stress granule proteins increased in the epithelia overlying the FF include eIF3A and eIF3B (32). We further compared the epithelia overlying FF to nonfibrotic control alveoli (Supplemental Figure 7). We found that the most increased pathways relate to translational control. Together with increased ER stress and translational control, the epithelia overlying the FF appear to play a vital role in fibroblast activation.

**Figure 8:**
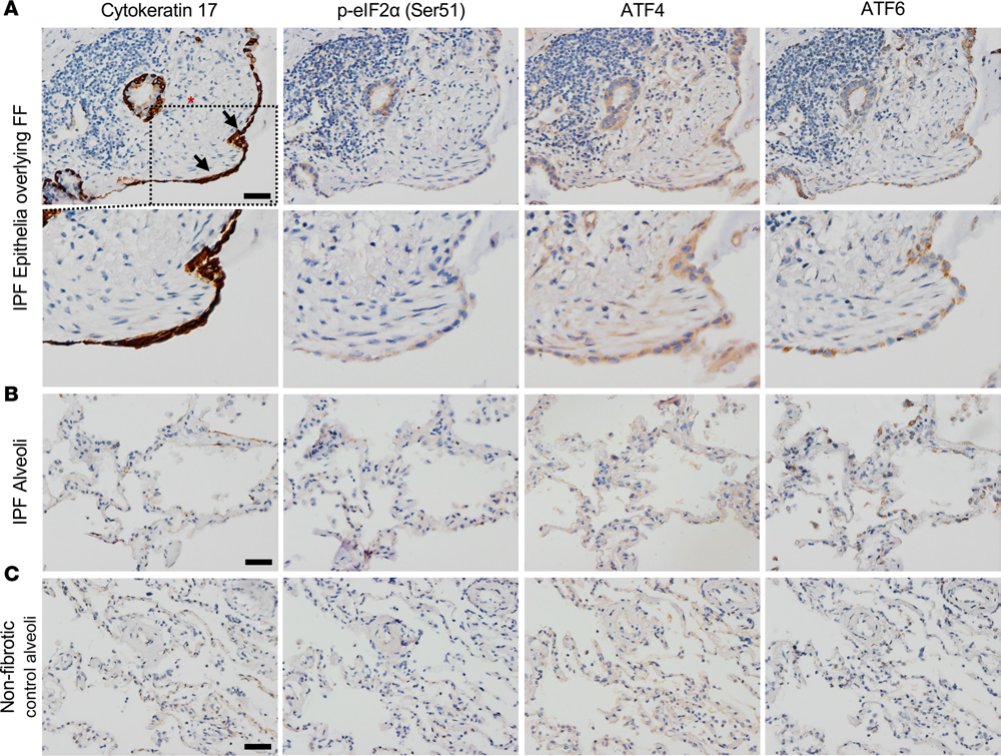
IPF epithelia overlying fibroblastic foci (FF) express stress markers. Representative images of IHC staining for cytokeratin 17, p-eIF2α (serine 51), ATF4, and ATF6 in (**A**) IPF epithelia (black arrows) overlying a fibroblastic foci (red asterisk), (**B**) IPF alveoli, and (**C**) nonfibrotic control alveoli. Scale bars: 50 μm.

To validate our spatial proteomic findings, we performed IHC on the IPF epithelia overlying the FF. We immunostained for cytokeratin 17 (marker of the epithelia overlying FF), p-eIF2α at Serine 51 and its downstream target ATF4 (33), and ATF6 (Figure 8). We found positivity of these markers, whereas these stains were less prominent in adjacent IPF alveoli or nonfibrotic control alveoli. Our results are consistent with an independent report that demonstrated that the epithelial cells overlying the FF are positive for ER stress markers ATF4 and ATF6 (28). Similar to previous findings, none of the 382 proteins that exhibited significant changes in the epithelia overlying FF were influenced by the *MUC5B* promoter variant (Figure 7E).

## Discussion

Our findings reveal that most proteomic changes in the lung associated with the *MUC5B* promoter variant are found in nonfibrotic unaffected lungs, particularly in the alveoli. In contrast, we found little evidence linking the *MUC5B* promoter variant to proteomic changes in IPF lungs. Given that the *MUC5B* promoter variant is the dominant risk factor for developing IPF, our findings suggest that enhanced expression of *MUC5B* in nonfibrotic unaffected lungs may induce early changes that predispose the lung to fibrosis by establishing a vulnerable bronchoalveolar epithelium. This is further supported by recent findings demonstrating that enhanced expression of Muc5b in either AT2 or airway cells alone does not drive fibrosis in vivo (34, 35). However, following bleomycin injury, Muc5b overexpression resulted in enhanced collagen deposition, honeycomb cyst formation, and mucus production (34, 35), suggesting that a second injury is needed to initiate the fibroproliferative response (6).

Extensive studies have identified that distal airways are the primary site of MUC5B expression. In control lungs, MUC5B-expressing cells are located within distal airways, which have an increased frequency in IPF lungs, particularly within honeycomb cysts. AT2 cells, however, do not express MUC5B protein (36). Another study confirmed that distal airways are the predominant site for MUC5B expression and noted that MUC5B expression is excluded from terminal bronchioles (37). Additionally, the *MUC5B* promoter variant is associated with increased MUC5B protein expression in IPF lung bronchioles (38) and single-cell RNA-seq data showed increased *MUC5B* mRNA expression primarily in alveolar cells as compared with the WT allele (39). The observation that alveolar cells ectopically express *MUC5B* when harboring the *MUC5B* promoter variant (39) combined with our findings showing that the promoter variant alters the proteomic signature of control alveoli highlights the need for further research into the role of *MUC5B* in alveolar biology.

Interestingly, IL-3 signaling is increased in the unaffected control alveoli in association with the *MUC5B* promoter variant. IL-3 is a cytokine produced by T lymphocytes and mast cells that stimulates the development of a variety of immune cells and plays a major role in inflammation (40). In experimental rodent models of acute lung injury, IL-3 knockout reduced proinflammatory mediators and neutrophil abundance (41). STAT5A is the most abundant MUC5B-associated protein in nonfibrotic alveoli, with increased expression of phosphorylation at Tyr694 in this region (Figure 1, C and D). This phosphorylation site is critical for STAT5A activation (15), and has been previously reported in IPF (42). In mammary alveolar cells, STAT5A is necessary and sufficient for the generation of alveolar luminal progenitor cells and mature alveoli (43). Thus, enhanced expression of *MUC5B* in nonfibrotic lungs may involve early changes that impact the alveoli.

A potential explanation for these results is the number of samples included in this study (*n* = 12 nonfibrotic control and *n* = 12 IPF specimens, with 6 harboring the *MUC5B* promoter variant per group). Prior to performing the research, we performed a power analysis on previous spatial proteomic data (8) and found that a sample size of 12 in each group would have 80% power to detect a fold change (FC) of 1.85, assuming a coefficient variable (CV) of 0.32 and FC of 2.5 for a CV of 0.5. Thus, the limited number of differentially expressed proteins in IPF samples is likely due to the dominant fibrotic signature masking the effect of *MUC5B*, and does not appear to be a function of the sample size. Another limitation is the reliance of IPA to identify the underlying biology of these regions (44). Further validation experiments are needed to confirm the identified pathways in these regions.

The unfolded protein response (UPR) is a type of ER stress that occurs when protein processing is disturbed, leading to the accumulation of misfolded proteins. ER stress is a substantial factor in lung fibrosis and has been previously associated with genetic risk variants (e.g., *SFTPC*) (45, 46). Given that the gain-of-function *MUC5B* promoter variant has also been associated with ER stress in epithelial cells (6, 34), *MUC5B* may contribute to this process. In fact, the most significantly increased MUC5B-associated protein in airways is UBX domain–containing protein 1 (UBXN1) (Figure 4E), a negative regulator of the UPR (47). In addition, the *MUC5B* promoter variant is associated with decreased expression of protein-folding proteins calreticulin (CALR) and calnexin (CANX) in airway epithelium (48, 49). Given that we found that CALR and CANX are increased in IPF airways, further work is needed to understand the mechanism by which the *MUC5B* promoter variant impacts ER stress proteins. A closer inspection of MUC5B-associated proteins in nonfibrotic control alveoli yielded several proteins associated with protein homeostasis (Figure 1B). For instance, BAG family molecular chaperone regulator 2 (BAG2) plays a prominent role in protein homeostasis by mediating protein refolding (50). In addition, 26S proteasome regulatory subunit-8 (PSMC5) and subunit-7 (PSMC2) are involved in protein homeostasis by degrading misfolded proteins (51). BAG2, PSMC5, and PSMC2 are MUC5B-associated proteins that are decreased in nonfibrotic control alveoli. Thus, it is plausible that enhanced expression of *MUC5B* may lead to ER stress and UPR in lung epithelia that predisposes the lung to fibrosis.

Herein, we are the first to our knowledge to determine the proteomic signature of the epithelia overlying FF. We found that the epithelia overlying FF clustered with alveolar proteins and are the most distant to airway groups (Figure 2), suggesting that they share properties with alveolar cells. Secondly, gene enrichment analysis of the uniquely expressed IPF epithelia overlying FF proteins show “cell response to stress” as the strongest category (Table 6) among the stress-related pathways (e.g., eIF2 signaling) (Table 7). How the epithelium becomes stressed is unknown. Other groups have shown that AT2 cells, upon injury or the ablation of alveolar type I (AT1) cells, differentiated into a prealveolar type 1 transitional cell state (PATS) that shares similarities with the epithelia overlying FF (52, 53). Consequently, the accumulation of PATS is associated with activated alveolar fibroblast and ECM deposition. Given that FF are lined with “stressed” epithelium, further work understanding *MUC5B*/ER stress/lung epithelium/mesenchymal crosstalk will likely enhance our understanding of FF development.

Our analysis revealed that IPF honeycomb cysts are defined by increased “remodeling of epithelial adherens junctions,” a pathway that was recently implicated in IPF distal airways using spatial transcriptomics (54). Adherens junctions regulate cell polarity, ECM deposition, and collective cell migration (55, 56). Collective cell migration is dysfunctional in IPF, resulting in increased migration compared with healthy counterparts (57, 58), a phenotype that was recapitulated by lung injury in vivo (59). Additionally, we found that the adhesion protein CD9 was differentially regulated by the *MUC5B* promoter variant in airways (Figure 4E). CD9 is important for regulating epithelial collective cell migration (60). Provided that ECM homeostasis is perturbed in IPF honeycomb cysts (8, 54) and that adherens junctions are influenced by ECM, this suggests a dynamic interplay between cell adhesion, adherens junctions, and ECM remodeling. Thus, fibrosis may be stimulated in an airway-centric manner following injury (e.g., smoking, *MUC5B*, etc.), which modulates airway cell adhesions and triggers ECM remodeling.

### Conclusion

By utilizing LCM-MS, we found that the *MUC5B* promoter variant, the strongest risk factor for IPF development, predominantly impacts the proteomic profiles of nonfibrotic lung tissue. We propose that enhanced *MUC5B* expression in lung epithelial cells may prime the epithelium through ER stress/UPR pathways for a secondary injury to initiate fibrosis. Furthermore, we found that the epithelium overlying the IPF FF exhibited cellular stress pathways. These findings underscore the role of the *MUC5B* promoter variant in priming the lung for fibrosis and emphasize the need to target ER stress pathways to mitigate IPF progression.

## Methods

### Sex as a biological variable.

Our study examined male and female human specimens, and sex was not considered as a biological variable.

### LCM.

We utilized an Olympus IX63 microscope integrated with Molecular Machinery Instruments (MMI) technologies to perform LCM. Fresh lung tissue was fixed in 4% formaldehyde for 48 hours at room temperature and then transferred into 70% ethanol prior to processing into formalin-fixed paraffin-embedded (FFPE) blocks. FFPE human lung tissue was sectioned at 5 μm and collected onto membrane slides (MMI, 50103) and stained with routine hematoxylin and eosin (H&E). Using MMI’s caplift technology, we captured regions of interest into tubes (MMI, 50204) and stored these samples at –80°C prior to processing for MS. Samples were stored in the freezer for a maximum of 3 months prior to processing.

### IHC.

FFPE lung tissue was sectioned at 5 μm and deparaffinized by submerging in a series of xylene and alcohol baths. Slides underwent antigen heat retrieval in either Universal HIER solution (Abcam, ab208572) or EDTA pH 8.0 for 20 minutes in a steamer and then allowed to cool for 20 minutes to room temperature. Slides were then treated with 3% hydrogen peroxide for 10 minutes and blocked for 1 hour with SuperBlock (Thermo Fisher Scientific, 37535). Primary antibody was added overnight in 10% SuperBlock solution. Antibodies against the following proteins were used: p-eIF2α (Cell Signaling Technology, 3398S; 1:50 in EDTA), cytokeratin 17 (Abcam, ab53707; 1:16,000 in HIER), ATF4 (Proteintech, 10835-1-AP; 1:16,000 in HIER), ATF6 (Abcam, ab227830; 1:1,500 in HIER), MUC5B (Novus Bio, NBP2-50522; 1:8,000 in HIER), p-STAT5A Tyr694 (Cell Signaling Technology, 9359S; 1:500 in HIER), CD9 (Abcam, ab2215; 1:80,000 in HIER), and MYOF (Invitrogen, PA5-53134; 1:8,000 in HIER). On the next day, we used a Novolink Polymer Kit (Leica Biosystems, RE7200-CE) following the manufacturer’s recommendations. Finally, we reacted the slides with DAB substrate (Abcam, ab64238) for 5 minutes followed by hematoxylin counterstaining and coverslipping.

### Microscopy and data analysis.

Bright-field images were acquired with an Olympus BX63 microscope using cellSens Dimension software. To count positive nuclei for p-STAT5A (Tyr694), we used ImageJ (NIH) to count the positive and negative nuclei using the Multi-Point Tool counter. For CD9 and MYOF quantification, we utilized the “Color Deconvolution2” plug-in to extract the H-DAB channel. We manually outlined the airways in the original image and overlaid them onto the H-DAB channel to determine the percentage of DAB positivity per region.

### Identification of histopathological features.

To define regions of interest, we collaborated with a lung pathologist who provided morphological assessments. Two serial sections were stained with anti–collagen I (to help demarcate FF) and anti-CK17 (to demarcate the epithelial cells overlying FF), aiding the identification of these structures on adjacent H&E sections. On the H&E stain, FF were histopathologically identified by their elongated, linear arrayed nuclei embedded in a pale staining matrix. FF had an epithelial lining to differentiate them from organizing pneumonia. Distal small airways (<2 mm) in IPF and nonfibrotic controls were surrounded by intact smooth muscle, while honeycomb cysts were found in regions of dense fibrosis lacking smooth muscle. For IPF alveoli, we captured regions adjacent to FF while avoiding blood vessels. For nonfibrotic control alveoli, we similarly captured alveoli while avoiding blood vessels. We collected volumes of 0.005–0.010 mm^3^ of tissue per sample by pooling multiple regions of interest from multiple serial sections.

### Sample processing for MS.

Laser-captured material was processed as previously described (7–9), with minor modifications that we highlight here. To achieve sample shearing, we utilized Covaris S220 and Sonolab 7.2 software at a setting of peak power of 200, duty factor of 20.0, cycle bursts of 200, with a duration of 50 seconds followed by a 10-second cool-down period (water temperature set to 4°C). This cycle was repeated 10 times for a total of 10 minutes. For sample digestion, we utilized 200 ng of trypsin in 25 μL of digestion buffer and incubated at 47°C for 2 hours. All other processing, digestion, and desalting steps were performed as previously described.

### MS acquisition.

Desalted peptides were adjusted to a protein concentration of 50 ng/μL with 0.1% formic acid (FA) in preparation for MS analysis. Digested peptides were loaded into autosampler vials and analyzed directly using a NanoElute liquid chromatography system coupled with a timsTOF SCP mass spectrometer. Peptides were separated on a 75 μm i.d. × 25 cm separation column packed with 1.6 μm C18 beads (IonOpticks) over a 90-minute elution gradient. Buffer A was 0.1% FA in water and buffer B was 0.1% FA in acetonitrile. Instrument control and data acquisition were performed using Compass Hystar (version 6.0) with the timsTOF SCP operating in parallel accumulation–serial fragmentation (PASEF) mode under the following settings: mass range 100–1700 *m*/*z*, 1/*k*/0, Start 0.7 V•s/cm^2^, and End 1.3 V•s/cm^2^. Ramp accumulation time was 166 ms, capillary voltage 4500 V, dry gas 8.0 L/min, and dry temp 200°C. The PASEF settings were 5 MS/MS scans (total cycle time, 1.03 s), charge range 0–5, active exclusion for 0.2 minutes, scheduling target intensity 20,000, intensity threshold 500, and collision-induced dissociation energy 10 eV.

### Data processing.

Data was searched using MSFragger via FragPipe v20.0 (http://fragpipe-analyst.nesvilab.org/). Precursor tolerance was set to ±15 ppm and fragment tolerance was set to ±0.08 Da. Data were searched against SwissProt restricted to *Homo* *sapiens* with added common contaminants (20,410 total sequences). Enzyme cleavage was set to semispecific trypsin for all samples. Fixed modifications were set as carbamidomethyl (C). Variable modifications were set as oxidation (M), oxidation (P) (hydroxyproline), Gln→pyro-Glu (N-term Q), and acetyl (Peptide N-term). Results were filtered to 1% FDR at the peptide and protein level. The data were then uploaded and processed with FragPipe-Analyst using LFQ settings, no normalization, *P*-value cutoff of 0.05, Perseus-type imputation, and FDR correction using Benjamini-Hochberg to acquire a dataset for further statistical processing.

### Statistics.

For the qualitative analysis, proteins were considered detected if they were detected in 80% or more of samples within each group. For quantitative analysis, we used a linear mixed model framework to test for differences between the *MUC5B* promoter variant or between region groups and account for repeated measures within subjects. Proteins were removed if they were undetected in greater than 24 samples. For the remaining proteins, we performed quantile normalization on the imputed matrix. To test for differential abundance in the quantitative analyses, we modeled the quantile normalized values by region group, including a random effect for subject using the R package lmerSeq (61). For the *MUC5B* promoter variant comparisons, data were subset to each region and tested for differentially abundant proteins between subjects with (GT) and without the risk variant (GG). We excluded results that had minimal variance (determined to be singular). Multiple testing correction was performed using Benjamini-Hochberg adjustment and statistical significance was set to an α level of 0.05. To identify up- or downregulated pathways, we applied IPA to the significant differential abundance results (62). For the reactome pathway analysis of the uniquely expressed proteins per region, we input the gene list into https://pantherdb.org/ and performed a PANTHER Overrepresentation test. We selected *Homo* *sapiens* as a reference list and the Annotation Data Set was set to “reactome pathways.” The test type was set to “Fisher’s exact” with correction set to “calculate false discovery rate.”

For IHC image analysis, we used GraphPad PRISM software for statistical analysis. We used an unpaired *t* test (2-tailed) for Figure 1F. For Figure 5, D and E, we used an ordinary 1-way ANOVA. A *P* value of less than 0.05 was considered significant.

### Study approval.

Patient demographics can be found in Supplemental Figure 8. All lung specimens met the criteria for IPF diagnosis following current guidelines (1). Patients provided written consent and samples were approved for research by the Colorado Multiple Institutional Review Board CB F490 (COMIRB 15-1147) and through the Lung Tissue Research Consortium (LTRC).

### Data availability.

The mass spectrometry proteomics data have been deposited to the ProteomeXchange Consortium via the PRIDE (63, 64) partner repository with the dataset identifier PXD058626. Values for all data points in graphs are reported in the Supporting Data Values file.

## Author contributions

JAH and DAS conceived and supervised the project. JAH, MM, and RB performed LCM-MS. JB procured lung specimens and CDC reviewed lung pathology. JAH, MM, and RZB analyzed data. JAH and NYL performed immunohistochemistry. JAH wrote the manuscript with input from all the authors (MM, RZB, RB, NYL, JB, CDC, JPH, JSK, CMM, KCH, IVY, and DAS).

## Supplementary Material

Supplemental data

Supplemental table 1

Supplemental table 2

Supplemental table 3

Supplemental table 4

Supporting data values

## Figures and Tables

**Table 1:**
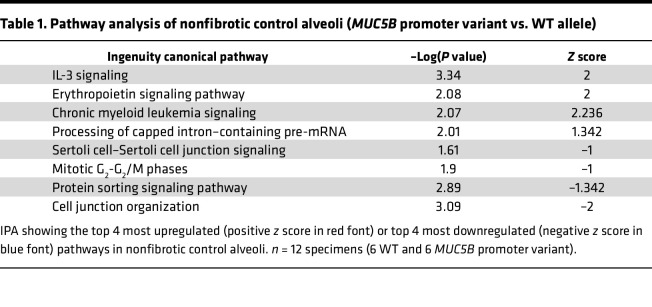
Pathway analysis of nonfibrotic control alveoli (*MUC5B* promoter variant vs. WT allele)

**Table 2:**
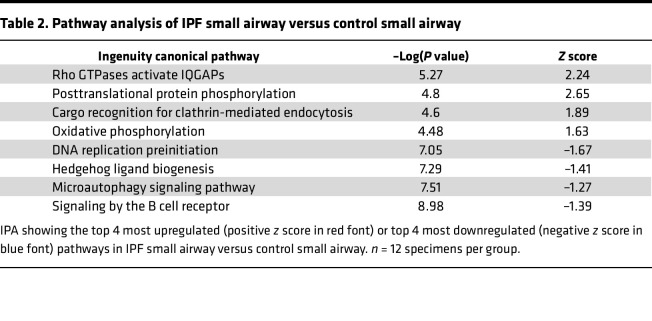
Pathway analysis of IPF small airway versus control small airway

**Table 3:**
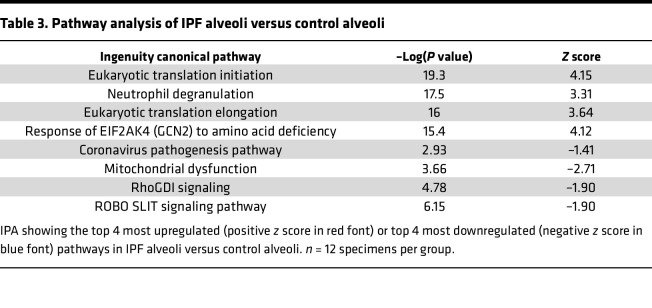
Pathway analysis of IPF alveoli versus control alveoli

**Table 4:**
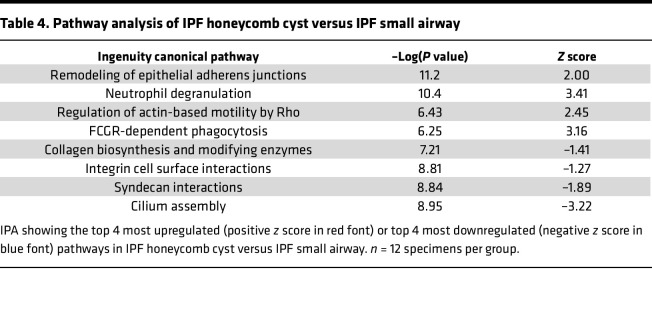
Pathway analysis of IPF honeycomb cyst versus IPF small airway

**Table 5:**
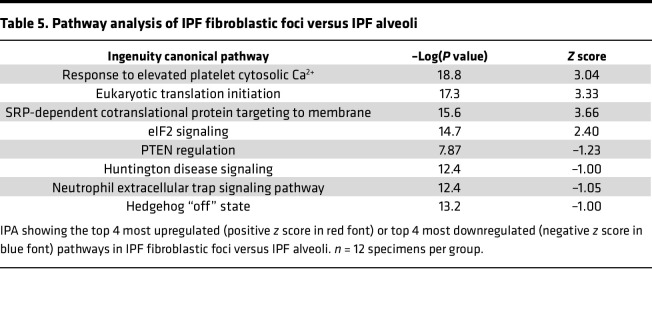
Pathway analysis of IPF fibroblastic foci versus IPF alveoli

**Table 6:**
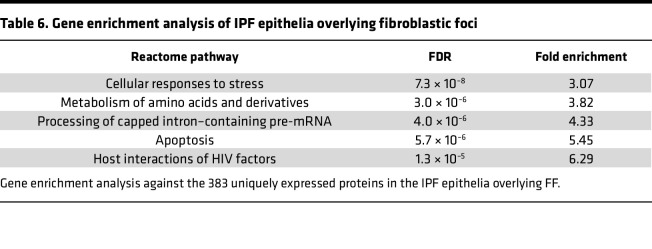
Gene enrichment analysis of IPF epithelia overlying fibroblastic foci

**Table 7:**
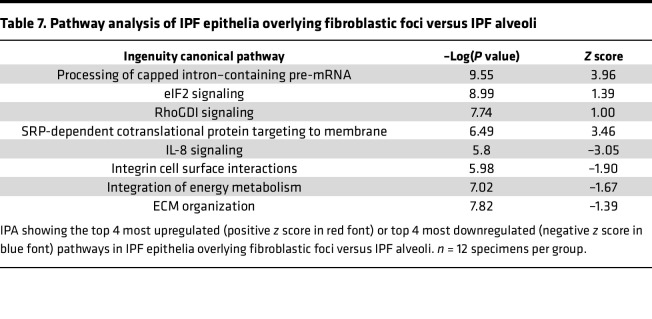
Pathway analysis of IPF epithelia overlying fibroblastic foci versus IPF alveoli

## Version 1 06/17/2025

In-Press Preview

## Version 2 07/22/2025

Electronic publication
