# Supplementary material for: The MUC5B promoter variant results in proteomic changes in the nonfibrotic lung
Source: JCI Insight. 2025 Jun 17;10(14):e189636. doi: 10.1172/jci.insight.189636 (PMC12288964; doi:10.1172/jci.insight.189636)
Supplement: Supplemental data [file jciinsight-10-189636-s108.pdf]

Supplemental Figure 1

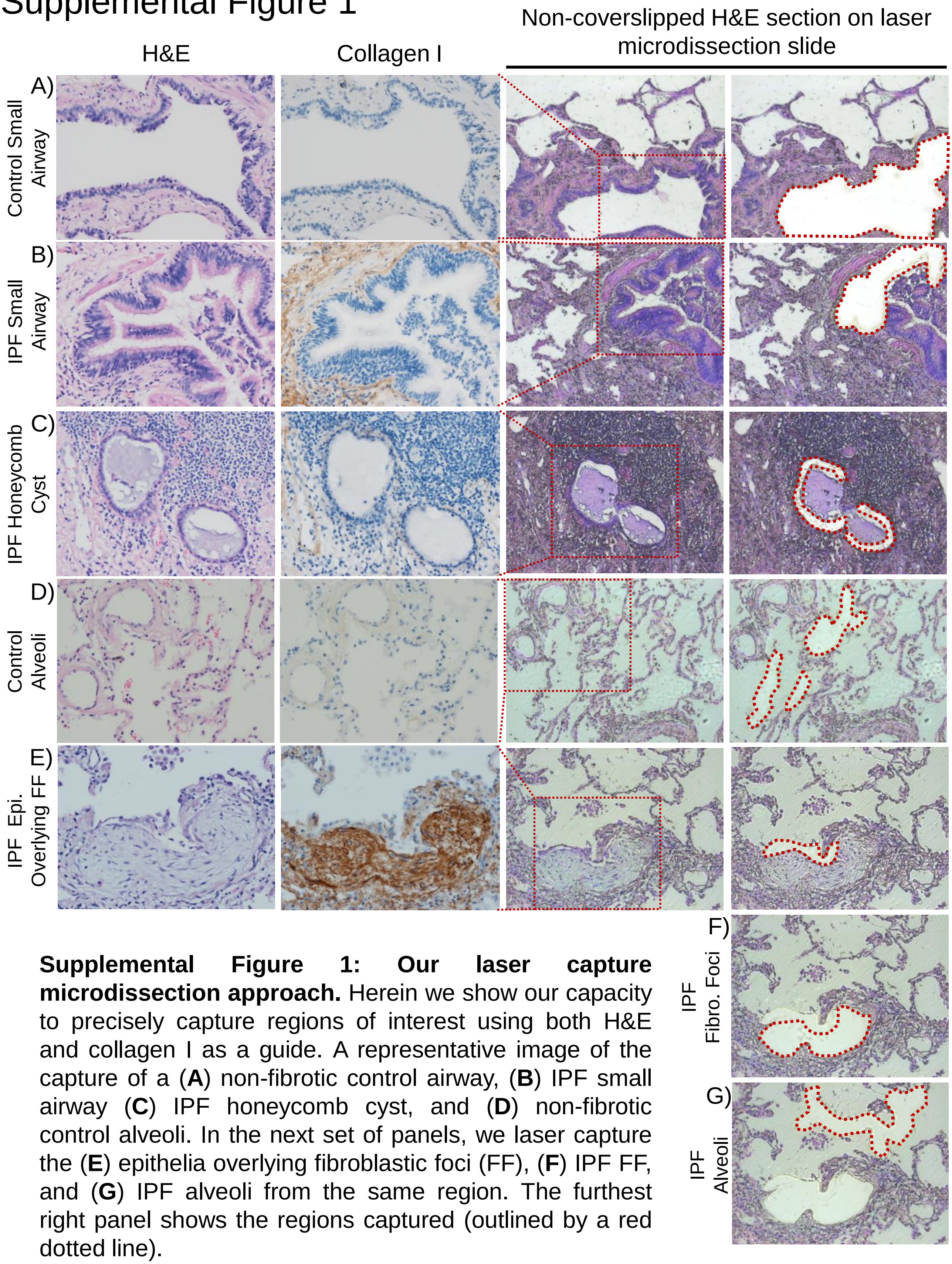

Supplemental Figure 1: Our laser capture microdissection approach. Herein we show our capacity to precisely capture regions of interest using both H&E and collagen I as a guide. A representative image of the capture of a (**A**) non-fibrotic control airway, (**B**) IPF small airway (**C**) IPF honeycomb cyst, and (**D**) non-fibrotic control alveoli. In the next set of panels, we laser capture the (**E**) epithelia overlying fibroblastic foci (FF), (**F**) IPF FF, and (**G**) IPF alveoli from the same region. The furthest right panel shows the regions captured (outlined by a red dotted line).

Supplemental Figure 2

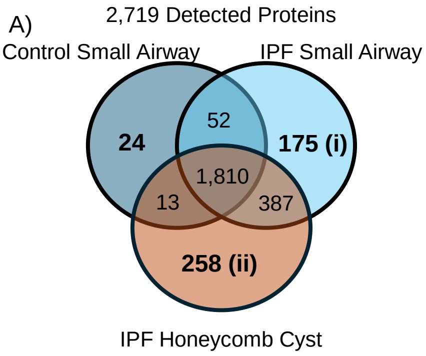

(i) IPF Small Airway
Gene Enrichment Analysis

Reactome Pathway	FDR	Fold-Enrichment
Anchoring of the basal body to the plasma membrane	7.8E-05	11.85
Cilium Assembly	4.7E-04	6.83

(ii) IPF Honeycomb Cysts
Gene Enrichment Analysis

Reactome Pathway	FDR	Fold-Enrichment
Membrane Trafficking	1.7E-03	3.06
Neutrophil degranulation	3.0E-03	3.2
Innate Immune System	1.2E-02	2.2
RHOQ GTPase cycle	4.9E-02	7.78

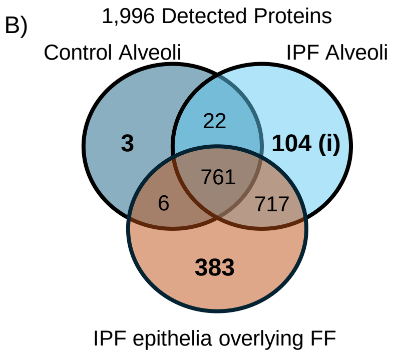

(i) IPF Alveoli
Gene Enrichment Analysis

Reactome Pathway	FDR	Fold-Enrichment
Regulation of insulin secretion	3.3E-05	20.11
Integration of energy metabolism	1.1E-04	14.66
G-protein activation	5.0E-03	32.69
Thromboxane signalling through TP receptor	6.0E-03	32.69
ADP signaling through P2Y purinoceptor 1	7.5E-03	32.69

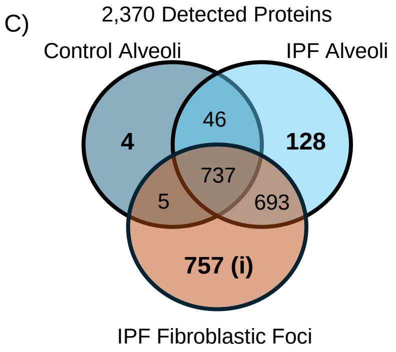

(i) IPF Fibroblastic Foci
Gene Enrichment Analysis

Reactome Pathway	FDR	Fold-Enrichment
Non-integrin membrane-ECM interactions	4.6E-07	6.67
Ribosomal scanning and start codon recognition	3.3E-06	6.22
Translation initiation complex formation	1.7E-05	5.78
Activation of the mRNA upon binding of the cap-binding complex and eIFs	1.9E-05	5.68
Collagen biosynthesis and modifying enzymbes	1.2E-05	5.48
Signaling by MET	1.0E-05	5.17
ECM Proteoglycan	1.1E-05	5.17
Collagen formation	3.5E-06	5.01

Supplemental Figure 2: Qualitative spatial analysis of IPF. Venn diagrams of detected proteins in (**A**) airway (**B**) alveolar, and (**C**) fibroblastic foci. A protein was considered detected if found in 80% of samples per group (left diagrams). A gene enrichment analysis of the uniquely expressed proteins using www.pantherdb.org is shown on the right. For the fibroblastic foci, we focused on the 50 most significantly regulated pathways of 357 arranged by fold-enrichment. N = 12 per group (n = 20 for fibroblastic foci).

Supplemental Figure 3

A)

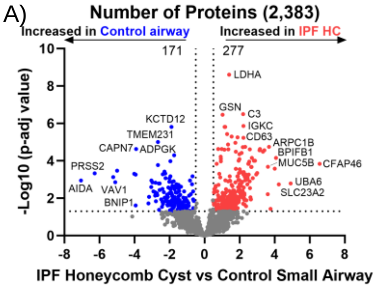

B) IPF Honeycomb Cyst vs Control Small Airway

Ingenuity Canonical Pathway	-Log(p-val)	Z-score
Neutrophil degranulation	24.0	4.16
Response to elevated platelet cytosolic Ca2+	16.4	4.49
Eukaryotic Translation Elongation	16.2	4.15
SRP-dependent cotranslational protein targeting to membrane	15.5	4.26
Extracellular matrix organization	4.5	-1.90
GP6 Signaling Pathway	4.54	-1.90
Assembly of collagen fibrils and other multimeric structures	4.75	-1.41
Collagen degradation	5.71	-1.67

MUC5B-associated

C)

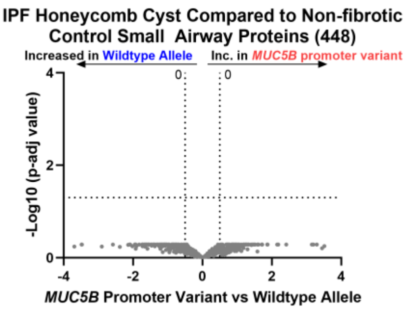

Supplemental Figure 3: IPF honeycomb cysts demonstrate deranged ECM remodeling. (**A & C**) Volcano plot comparing IPF honeycomb cysts and non-fibrotic control small airways (A) for the number of proteins analyzed and (C) the subset of significantly changed proteins from (A) comparing for the *MUC5B* promoter variant, showing the negative natural log of p-adjusted values plotted against the base 2 log (fold change) for each protein. (**B**) Ingenuity pathway analysis showing the top 4 most upregulated (positive Z-score in red font) or top 4 most downregulated (negative Z-score in blue font) pathways per comparison. N = 12 per group.

Supplemental Figure 4

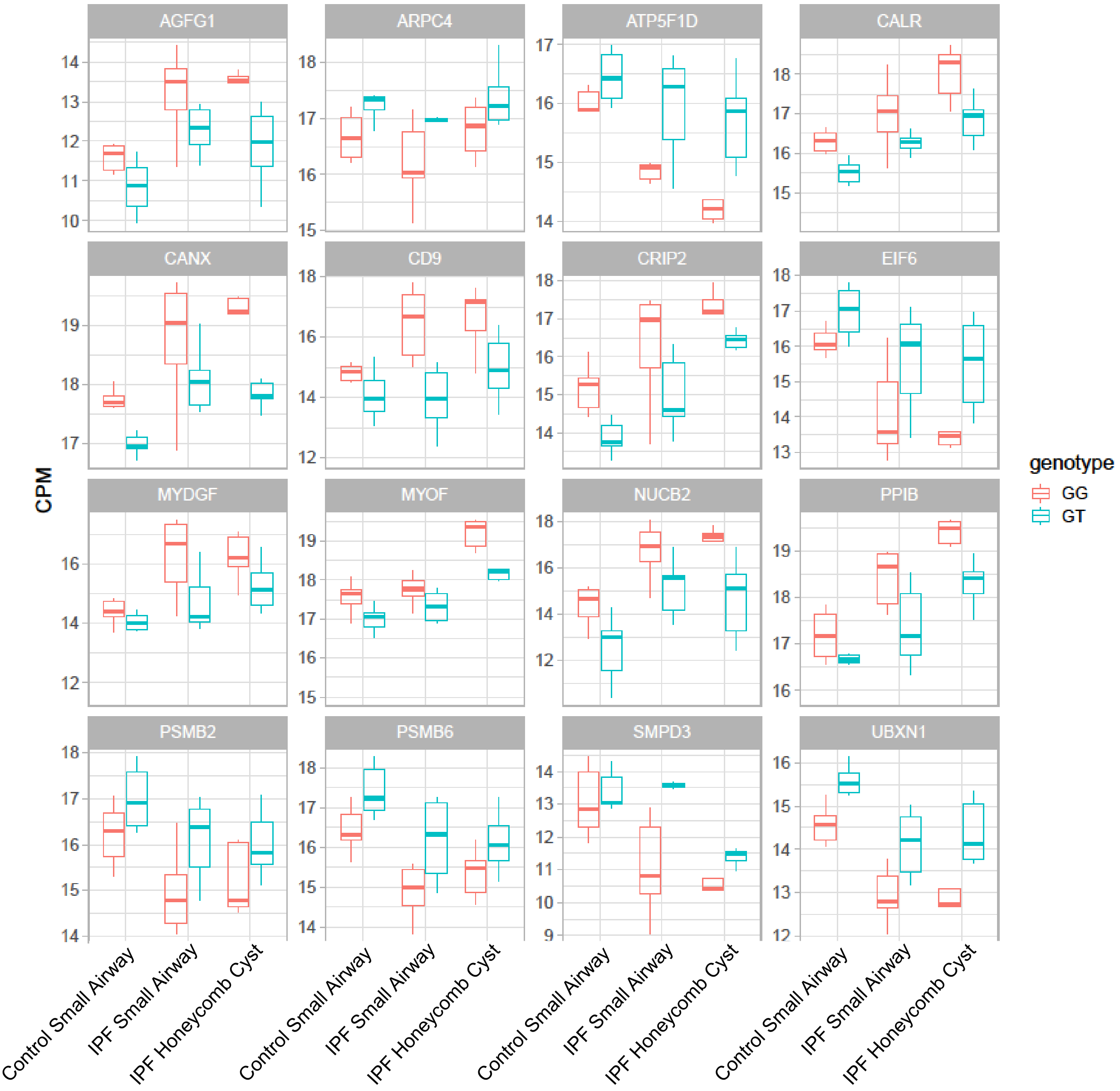

Supplemental Figure 4: Impact of *MUC5B* promoter variant on the proteomic profiles of airways. Shown is the effect of *MUC5B* promoter variant (GT) on control small airway, IPF small airway, or IPF honeycomb cyst (n = 6 per group). Although underpowered per group individually, our combined analysis when controlling for disease identified 11 significantly changed proteins when comparing for *MUC5B* promoter variant (original Figure 4E).

Supplemental Figure 5

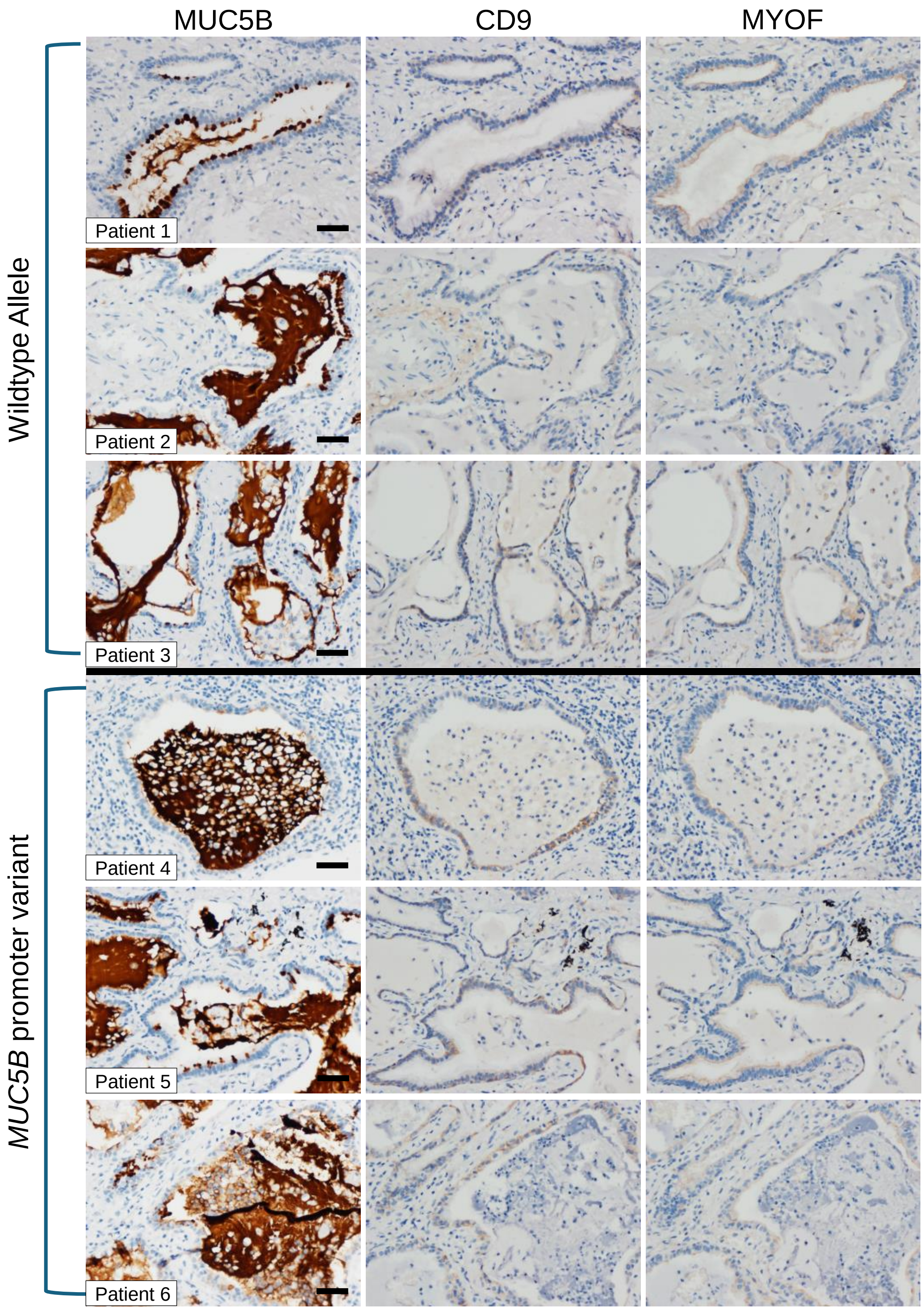

Supplemental Figure 5: Expression of CD9 and MYOF in IPF honeycomb cyst: Immunohistochemistry against MUC5B, CD9, and MYOF in 6 IPF specimens (3 wildtype allele and 3 *MUC5B* promoter variant). Scale bar represents 50 microns.

Supplemental Figure 6

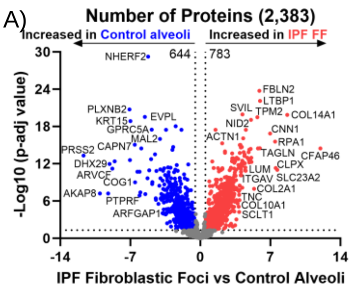

B)

IPF Fibroblastic Foci vs Control Alveoli

Ingenuity Canonical Pathway	-Log(p-val)	Z-score
Eukaryotic Translation Initiation	41	5.76
SRP-dependent cotranslational protein targeting to membrane	35.7	6.07
Eukaryotic Translation Elongation	35.1	5.37
Eukaryotic Translation Termination	34.2	5.286
Processing of Capped Intron-Containing Pre-mRNA	9.46	-1.02
mTOR Signaling	11.7	-1.39
Mitochondrial dysfunction	13.8	-2.32
Huntington's Disease Signaling	14	-2.00

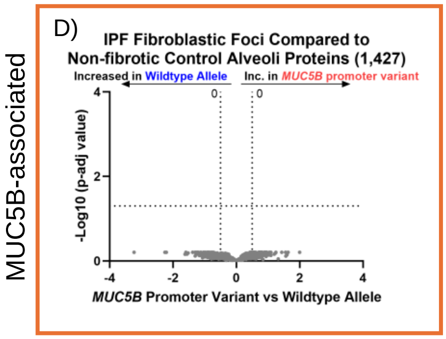

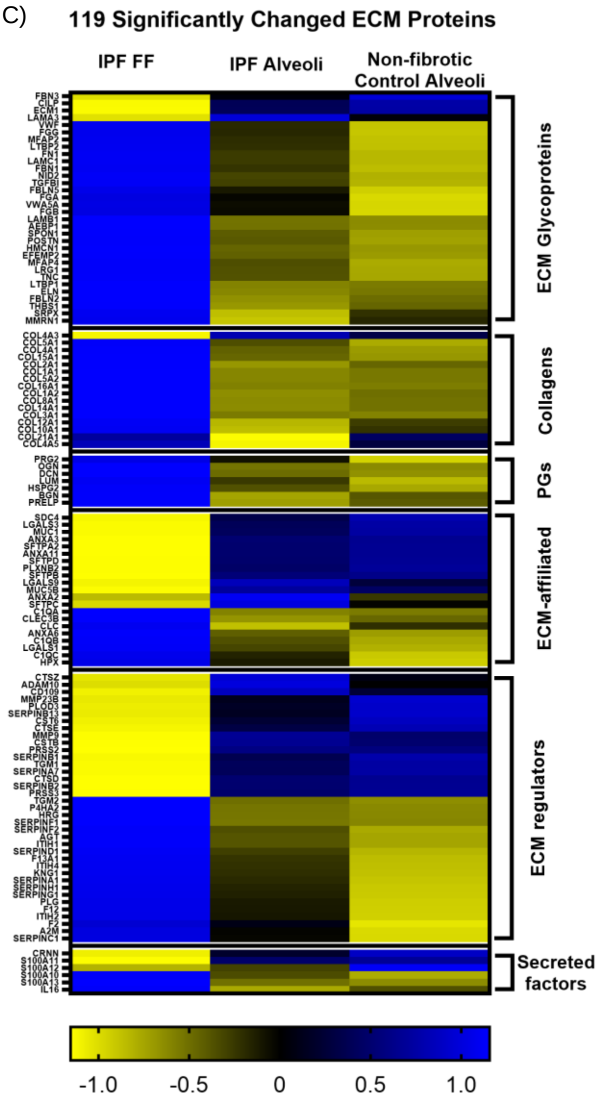

Supplemental Figure 6: IPF fibroblastic foci are defined by increased translational control. (**A & D**) (A)Volcano plot comparing IPF FF and non-fibrotic control alveoli and, (D) the subset of significantly changed proteins from (A) when comparing the *MUC5B* promoter variant, showing the negative natural log of p-adjusted values plotted against the base 2 log (fold change) for each protein. (**B**) Ingenuity pathway analysis showing the top 4 most upregulated (positive Z-score in red font) or top 4 most downregulated (negative Z-score in blue font) pathways. (**C**) A heatmap displaying Z-scores on the significantly changed extracellular matrix proteins categorized by their groups comparing IPF FF, IPF alveoli, and non-fibrotic control alveoli. N= 12 per group (n = 20 FF, balanced for the *MUC5B* promoter variant).

Supplemental Figure 7

A)

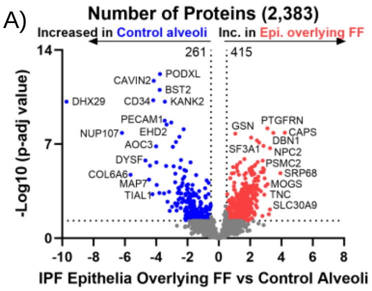

B)

IPF Epi. Overlying FF vs Control Alveoli

Ingenuity Canonical Pathway	-Log(p-val)	Z-score
Eukaryotic Translation Initiation	45.2	6.30
SRP-dependent cotranslational protein targeting to membrane	41.2	6.27
Eukaryotic Translation Elongation	40	6.25
Neutrophil degranulation	39.1	3.09
Coronavirus Pathogenesis Pathway	8.89	-3.00
Gene and protein expression by JAK-STAT signaling after IL-12 stimulation	9.53	-1.16
Regulation of eIF4 and p70S6K Signaling	13.8	-1.00
mTOR Signaling	14.4	-2.11

MUC5B-associated

C)

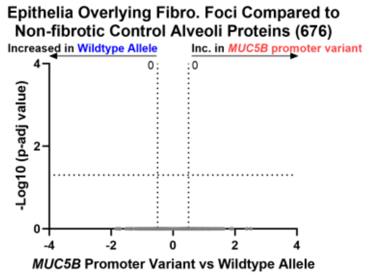

Supplemental Figure 7: IPF epithelia overlying fibroblastic foci demonstrate increased translational control. (**A & C**) Volcano plot comparing (A) IPF epithelia overlying FF and non-fibrotic control alveoli, and (C) the subset of significantly changed proteins in (A) assessed for the *MUC5B* promoter variant, showing the negative natural log of p-adjusted values plotted against the base 2 log (fold change) for each protein. (**B**) Ingenuity pathway analysis showing the top 4 most upregulated (positive Z-score in red font) or top 4 most downregulated (negative Z-score in blue font) pathways per comparison. N = 12 per group.

Supplemental Figure 8

Control or IPF	MUC5B Genotype	Age	Sex	Smoking Status	Ethnicity/Race
Control	GG	69	Male	Former	Non-Hispanic White
Control	GG	47	Male	Current	Unknown
Control	GG	71	Male	Former	Non-Hispanic White
Control	GG	62	Female	Never	Unknown
Control	GG	57	Male	Never	European decent
Control	GG	79	Female	Never	Non-Hispanic White
Control	GT	67	Female	Unknown	Non-Hispanic White
Control	GT	74	Male	former	Non-Hispanic White
Control	GT	49	Female	Former	Non-Hispanic White
Control	GT	66	Male	Former	Non-Hispanic White
Control	GT	66	Male	Former	Non-Hispanic White
Control	GT	63	Male	Former	Non-Hispanic White
IPF	GG	65	Male	Former	Non-Hispanic White
IPF	GG	59	Female	Never	Non-Hispanic White
IPF	GG	66	Male	Former	Non-Hispanic White
IPF	GG	68	Male	Former	Non-Hispanic White
IPF	GG	52	Male	Former	Non-Hispanic White
IPF	GG	68	Female	Former	Non-Hispanic White
IPF	GT	66	Male	Never	Non-Hispanic White
IPF	GT	71	Female	Former	Non-Hispanic White
IPF	GT	69	Female	Former	Non-Hispanic White
IPF	GT	68	Female	Never	Non-Hispanic White
IPF	GT	68	Male	Former	Non-Hispanic White
IPF	GT	65	Male	Never	Non-Hispanic White

Supplemental Figure 8: Patient demographics. For the *MUC5B* promoter variant genotype, our wildtype allele is represented as GG whereas all the specimens (control and IPF) that harbor the MUC5B promoter variant are heterozygous (GT).
